# Supplementary material for: Functional Regulation of ZnAl-LDHs and Mechanism of Photocatalytic Reduction of CO2: A DFT Study
Source: Molecules. 2023 Jan 11;28(2):738. doi: 10.3390/molecules28020738 (PMC9863086; doi:10.3390/molecules28020738)
Supplement: Supplementary file 1 [file molecules-28-00738-s001.zip › molecules-2092955-supplementary.pdf]

Table S1. The lattice and geometric parameters of ZnAl-NO_3-LDHs.

Method	Lattice parameter (Å)			Bond length (Å)			Bond angle (°)	
	a	b	c	Zn-O	Al-O	O-H	O-Zn-O	O-Al-O
PBE	3.13	3.13	8.75	2.211	1.825	0.982	75.41	84.74
PBE+vdW	3.08	3.08	8.86	2.084	1.882	0.981	78.36	87.31
Exptl [a]	3.08	3.08	8.86	2.082	1.854	0.967	86.43	86.39
Dmol3 [b]	3.10	3.10	8.66	2.028	2.014	0.990	79.27	79.95
revPBE-vdW [c]	3.18	3.18	8.78	-	-	-	-	-

[a] The parameters are from ref. [31]. [b] The parameters are from ref. [24]. [c] The parameters are from ref. [40].

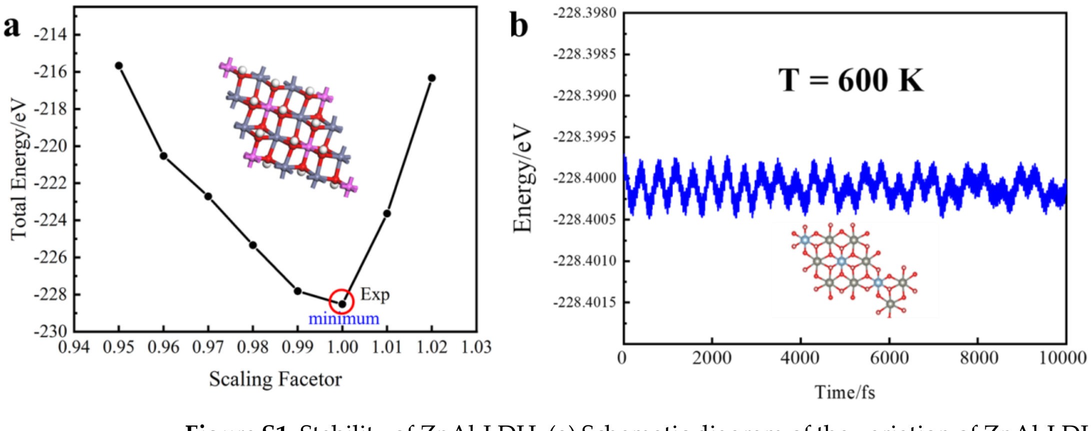

Figure S1. Stability of ZnAl-LDH. (**a**) Schematic diagram of the variation of ZnAl-LDH energy with lattice parameters, where Exp corresponds to the lattice parameters obtained experimentally on ZnAl-LDH in ref. [34]; (**b**) Variation of energy of ZnAl-LDH at 600 K with time by AIMD simulation.
